# Kinetics and Mechanism Study of Competitive Inhibition of Jack-Bean Urease by Baicalin

**DOI:** 10.1155/2013/879501

**Published:** 2013-10-01

**Authors:** Lirong Tan, Jiyan Su, Dianwei Wu, Xiaodan Yu, Zuqing Su, Jingjin He, Xiaoli Wu, Songzhi Kong, Xiaoping Lai, Ji Lin, Ziren Su

**Affiliations:** ^1^School of Chinese Materia Medica, Guangzhou University of Chinese Medicine, Guangzhou, Guangdong 510006, China; ^2^Dongguan Mathematical Engineering Academy of Chinese Medicine, Guangzhou University of Chinese Medicine, Dongguan, Guangdong 523808, China

## Abstract

Baicalin (BA) is the principal component of Radix Scutellariae responsible for its pharmacological activity. In this study, kinetics and mechanism of inhibition by BA against jack-bean urease were investigated for its therapeutic potential. It was revealed that the IC_50_ of BA against jack-bean urease was 2.74 ± 0.51 mM, which was proved to be a competitive and concentration-dependent inhibition with slow-binding progress curves. The rapid formation of initial BA-urease complex with an inhibition constant of *K*_*i*_ = 3.89 × 10^−3^ mM was followed by a slow isomerization into the final complex with an overall inhibition constant of *K*_i_* = 1.47 × 10^−4^ mM. High effectiveness of thiol protectors against BA inhibition indicated that the strategic role of the active-site sulfhydryl group of the urease was involved in the blocking process. Moreover, the inhibition of BA was proved to be reversible due to the fact that urease could be reactivated by dithiothreitol but not reactant dilution. Molecular docking assay suggested that BA made contacts with the important activating sulfhydryl group Cys-592 residues and restricted the mobility of the active-site flap. Taken together, it could be deduced that BA was a competitive inhibitor targeting thiol groups of urease in a slow-binding manner both reversibly and concentration-dependently, serving as a promising urease inhibitor for treatments on urease-related diseases.

## 1. Introduction

Urease (urea amidohydrolases, EC 3.5.1.5) is a thiol-rich and nickel-dependent metalloenzyme that can catalyze the hydrolysis of urea, thereby producing ammonia and carbamate [1]. Urease can be synthesized by numerous organisms, including plants, bacteria, algae, fungi, and invertebrates, and it also occurs in soils as a soil enzyme [2]. Ni^2+^ ions and the sulfhydryl group, especially the multiple cysteinyl residues in the active site of the enzyme, are essential for the catalytic activity of all ureases. Importantly, ureolytic activity of bacteria, such as *Proteus mirabilis*, *Klebsiella pneumoniae*, *Staphylococcus* spp., *Salmonella* sp., and *Ureaplasma urealyticum*, is a vital virulence factor implicated in the pathogenesis of many clinical conditions, including pyelonephritis, hepatic coma, peptic ulceration, and formation-infection-induced urinary stones [3, 4]. The pathogenesis is due to the consequent of urea hydrolysis, which results in a pH increase (up to ca. 9.2) and the toxicity of the released ammonia and of its derivatives [3]. Moreover, urease activity has been defined as the vital virulence factor for *Helicobacter pylori* infection, which enables this bacterium to survive at low pH of the stomach during colonization causing peptic ulcers and stomach cancer [5]. Therefore, strategies based on urease inhibition are now considered as the first line of treatment for infections caused by urease-producing microorganisms. 

**Figure 1:**
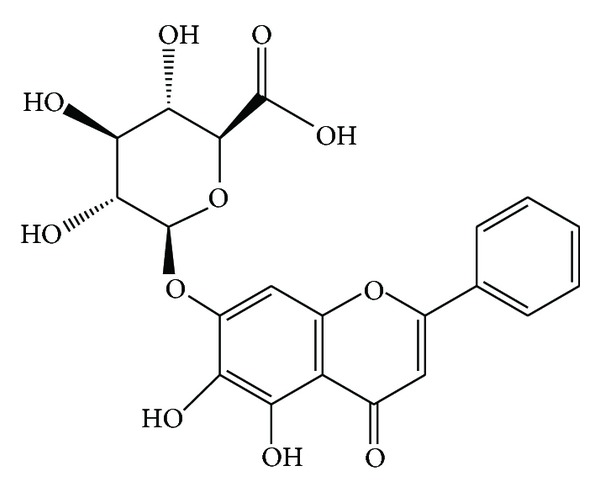
Chemical structure of baicalin.

 Flavonoids, the derivates of 2-phenyl-1,4-benzopyrone, were found to be efficient inhibitors against urease. For example, quercetin glycosides could inhibit jack-bean urease activity at micromolar level [6], and hesperetin [7] inhibited 57% of the urease activity of *Helicobacter pylori* at 0.3 mg/mL. Radix Scutellariae, known as “Huang-Qin” in Chinese, is originated from the dried root of *Scutellaria baicalensis* Georgi (family of Labiatae). Its therapeutic functions in Chinese medicine are to remove *damp-heat*, and quench *fire,* to counteract *toxicity*, to arrest bleeding, and to prevent abortion [8]. It is not only widely used in traditional Chinese herbal medicine, but it is also used as a food additive. Baicalin (BA, C_21_H_18_O_11_, shown in Figure 1), a flavonoid glucuronide, is one of the major bioactive compounds of Radix Scutellariae and is commercially available in hair shampoo. It has also been demonstrated that BA has promising activities in diverse areas such as anti-inflammation [9], antioxidant [10], antibacterial [11], and antiallergic properties [12] and anticancer activities [13], as well as neurofibril disruption [14]. Researches have revealed the antimicrobial activity of BA against *Helicobacter pylori *[15, 16] and *Chlamydia trachomatis in vitro *[17] and the potential ability on *Helicobacter-pylori*-induced gastric inflammation [18]. It was also reported that BA showed wide range of enzymes inhibitory influences on renin, angiotensin-I-converting enzyme, aldose reductase, and sialidase [19, 20]. Therefore, BA is expected to exert inhibitory properties against urease, through counteracting the undesirable effects brought about by activated urease, although the urease-inhibiting properties of BA have not been well characterized.

The aim of this study is to investigate the inhibitory effect of BA on commercial jack-bean (*Canavalia ensiformis*) urease. Attempts were made to elucidate the kinetics and mechanism of inhibition based on the reaction with thiols, in order to clarify the role of the urease active-site sulfhydryl group in the inhibition by BA. 

## 2. Materials and Methods

### 2.1. Materials

 Baicalin (C_21_H_18_O_11_, CAS number: 21967-41-9), urea (molecular biology reagent), D,L-dithiothreitol (DTT), glutathione (GSH), L-cysteine (L-cys), boric acid, and sodium fluoride (NaF) were purchased from Sigma Aldrich. Urease (from jack beans, type III, nominal activity 40.3 units/mg, solid) was also from Sigma Aldrich, of which one unit of urease activity is defined as the amount of enzyme needed to liberate 1.0 *μ*mol of NH_3_ from urea per min at pH 7.0 at 25°C. Other chemicals were obtained from Guangzhou Chemical Reagent Factory (China). All reagents were of analytical grade. Phosphate buffer (PBS, 20 mM, pH 7.0) was prepared by adjusting pH of phosphoric (V) acid with NaOH. 2 mM EDTA was added to all enzyme-containing solutions.

### 2.2. Determination of *K*_*M*_ and *v*_max_

The Michaelis constant *K*_*M*_ and the maximum velocity *v*_max⁡_ in the absence of the inhibitor were determined by measuring the initial reaction velocities at different urea concentrations ranging from 0.4 mM to 10 mM. The values were obtained by applying nonlinear regression to the Michaelis-Menten equation.

### 2.3. Standard Urease Activity Assay

 The standard urease assay mixture contained 50 mM urea in 20 mM phosphate buffer (pH 7.0) containing 2 mM EDTA. After addition of the enzyme-containing solution of 0.25 mg/mL urease, the assay ran for 20 min, and the enzyme activity was determined by measuring the concentration of the ammonia released in the reaction mixture. For ammonia measurement, aliquots were withdrawn from the reaction mixtures, and the ammonia was determined at 595 nm by a spectrophotometric according to the modified Berthelot (phenol-hypochlorite) method [21] at ambient temperature. 

### 2.4. Inactivation of Urease by BA

Urease solutions mixed with serial concentrations of BA (0.70–5.25 mM) were incubated at 37°C for 20 min, which contained 0.25 mg/mL urease, 20 mM phosphate buffer (pH 7.0), and 2 mM EDTA. The initial time of incubation was defined as the moment once the enzyme and the inhibitor were mixed. After appropriate period of time, aliquots from the incubation mixture were transferred into the standard assay mixtures for urease residual activity determination. The activity of uninhibited urease was defined as the control activity of 100%.

### 2.5. Reaction Progress Curves Monitoring

The reaction progress was studied in the absence or presence of BA using the following two procedures.

1. *Unpreincubated System*. The progress curves were determined by the reactions directly initiated by the addition of the enzyme into the reaction mixtures containing different concentrations of BA (1.75, 2.75, and 3.75 mM).
2. *Preincubated System*. The enzyme was preincubated with BA for 20 min first, and the reaction was then initiated by addition of urea solution into the reaction preincubation mixtures containing different concentrations of BA (1.75, 2.75, and 3.75 mM).

Urease activities in both procedures were determined as described in Section 2.3. A curve-fitting computer program was employed to fit the experimental points to the integrated equation describing slow-binding inhibition progress curves [22]:

(1) $$\begin{array}{c} P\left(t\right)=v_{s}t+\frac{\left(v_{o}-v_{s}\right)\left(1-e^{-k_{\text{app}}t}\right)}{k_{\text{app}}}, \end{array}$$

where *P* is the amount of product accumulated at time *t* after initiation of the reaction. *v*_*o*_ and *v*_*s*_ are the reaction initial and steady-state velocities, respectively, and *k*_app_ denotes the apparent first-order velocity constant for interconversion between *v*_*o*_ and *v*_*s*_.

### 2.6. Urease Protection against BA Inactivation

 Urease protection studies were carried out as follows. Urease was first preincubated with different protectors for 20 min. Then, samples of the protected urease were incubated with 2.50 mM BA for additional 20 min. The urease activity was assayed upon incubation of the mixture. For protection by thiols, the applied thiol-containing compounds (L-cys, GSH, and DTT) were of 1.95 to 11.67 mM. For protection by boric acid and fluoride, the enzyme was preincubated with 2.50 mM boric acid and 2.50 mM sodium fluoride.

### 2.7. BA-Thiol-Urease Interaction Test

The incubation mixtures contained urease solution, BA, and dithiol (DTT) or monothiols (L-cys and GSH). The components of the incubation mixture were mixed according to the following three procedures.

- Urease was added to the mixture after a 20 min contact of BA with the thiol.
- BA was added to the mixture after a 20 min contact of urease with thiol.
- Thiol was added to the mixture after a 20 min contact of urease with BA.

The complete mixture was mixed thoroughly and incubated for additional 5, 10, 20, and 40 min. Then, urease activity assay was determined as described in the inactivation of urease by BA.

### 2.8. Reactivation of BA-Inactivated Urease

The reactivation of inactivated urease was studied in two ways: by using DTT and by multidilution in the reaction mixture containing urea.

1. After a 20 min preincubation of urease with BA (3.75 mM), the mixture was further incubated with DTT (final concentration of 3.75 mM) for 120 min. The activity of urease was determined before and after the addition of DTT.
2. BA (3.75 mM) was preincubated for 10 and 20 min, respectively, with the enzyme to establish the equilibrium: *E* + *I*⇔*EI*⇔EI*, and, then, the preincubation mixture was diluted 50 folds into the reaction mixture. After appropriate period of time, aliquots were withdrawn, and the amount of ammonia was determined.

### 2.9. Molecular Docking

The automated docking studies were carried out using Auto-Dock version 4.0 as implemented through the graphical user interface AutoDock Tools (ADT 1.5.2). The three-dimensional crystal structure of jack-bean urease (PDB code: 3LA4) was obtained from the RCSB Protein Data Bank, whose resolution was 2.05 Å. The required actions were to remove water molecules from the protein, add all hydrogen atoms, calculate Gasteiger charges, and merge nonpolar hydrogen atoms to carbon atoms. The standard 3D structure (PDB format) of BA was obtained with chem3D Ultra 8.0 software. The PDB files were further transferred to PDBQT files with AutoDock Tools. The three-dimensional results were created by the PyMol molecular graphics system [23].

 The cubic grid box of 60 Å size (*x*, *y*, *z*) with a spacing of 0.5 Å and grid maps were built. The center of the grid was set to the average coordinates of the two Ni^2+^ ions. The Lamarckian genetic algorithm (LGA) was selected as the search algorithm.

The Lamarckian job consisted of 100 runs. Default settings were used with an initial population of 150 randomly placed individuals, a maximum number of 2.5 × 10^6^ energy evaluations, and a maximum number of 2.7 × 10^4^ generations. A mutation rate of 0.02 and a crossover rate of 0.8 were chosen. Van der Waals and hydrogen bonding were included in the calculated nonbonded energy. Results differing by less than 0.5 Å in positional root-mean-square deviation (RMSD) were clustered together, and the results of the most favorable free energy of binding were chosen as the resultant complex structures.

## 3. Results and Discussion

### 3.1. Urease Inhibition Assays

**Figure 2:**
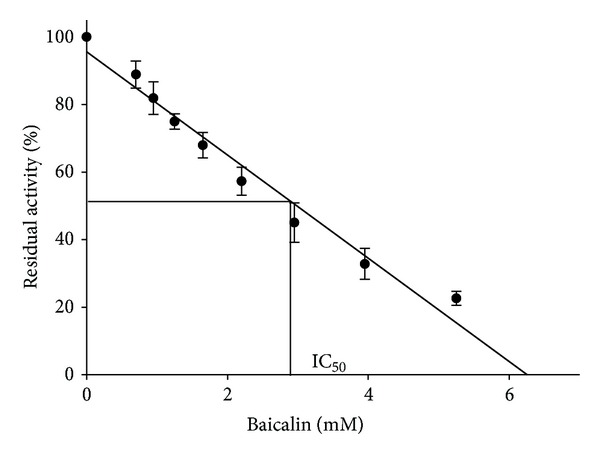
Dependence of residual activity versus concentration of BA. The results are expressed as means ± SD of the data from triplicate tests.

Data from Figure 2 depicted enzyme residual activity as a function of BA concentration. The linear function for this relation is a good-enough approximation (*R* ^2^ = 0.97). The obtained IC_50_ value was 2.74 ± 0.51 mM, where the IC_50_ indicated the BA concentration that could descend the activity of 10 U/mL urease to 50%.

### 3.2. Kinetics of Urease Inactivation by BA

**Figure 3:**
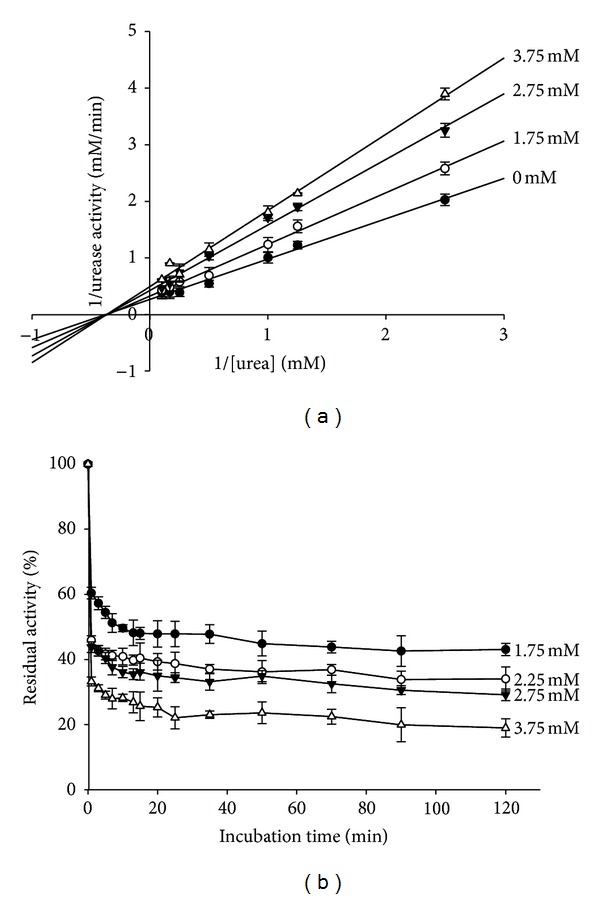
(a) Lineweaver-Burk plot of the reciprocal of urease activity versus reciprocal of substrate concentration in the absence (*⚫*) and presence of 3.75 mM (△), 2.75 mM (*▼*), and 1.75 mM (○) of BA. (b) Dependence of residual activity versus preincubation time with BA. Concentration of BA (mM) is numerically given. Each value represents the mean ± SD from three independent experiments.

Enzyme kinetics was determined in the absence and presence of various concentrations of BA. *K*_*M*_ and *v*_max⁡_ of ureolytic reaction by applying nonlinear regression to the Michaelis-Menten equation were 2.52 ± 0.12 mM and 3.64 ± 0.11 mM/min, respectively. As the Lineweaver-Burk plots for BA showed in Figure 3(a), *K*_*M*_ value did not significantly change in the presence of BA, while the *v*_max⁡_ value decreased as the BAs concentration increased, indicating that BA may be a noncompetitive mechanism of inhibition.

 On the other hand, our data indicated a slow-binding inhibition relationship of enzyme activity *versus* preincubation time [24, 25], which indicated the total urease activity in the free form and in the form of being bound in the urease-inhibitor complexes *EI* and EI*. It was clear in Figure 3(b) that increasing the preincubation time resulted in a decrease of urease activity. The activity descended rapidly at the beginning until the equilibrium between urease (*E*), inhibitor (*I*), and urease-inhibitor complexes (*EI*) and (EI*) (*E* + *I*⇔*EI*⇔EI*) was achieved, which was characterized by the constant urease activity, since the slow-binding effect would not be revealed unless the enzyme interacted with the inhibitor for sufficient time. Otherwise, it would lead to a misinterpretation as a noncompetitive type if determined by the initial reaction rates method. Hence, the progress curves analysis was employed to confirm the slow-binding model of urease inactivation by BA.

### 3.3. Progress Curves Analysis

**Figure 4:**
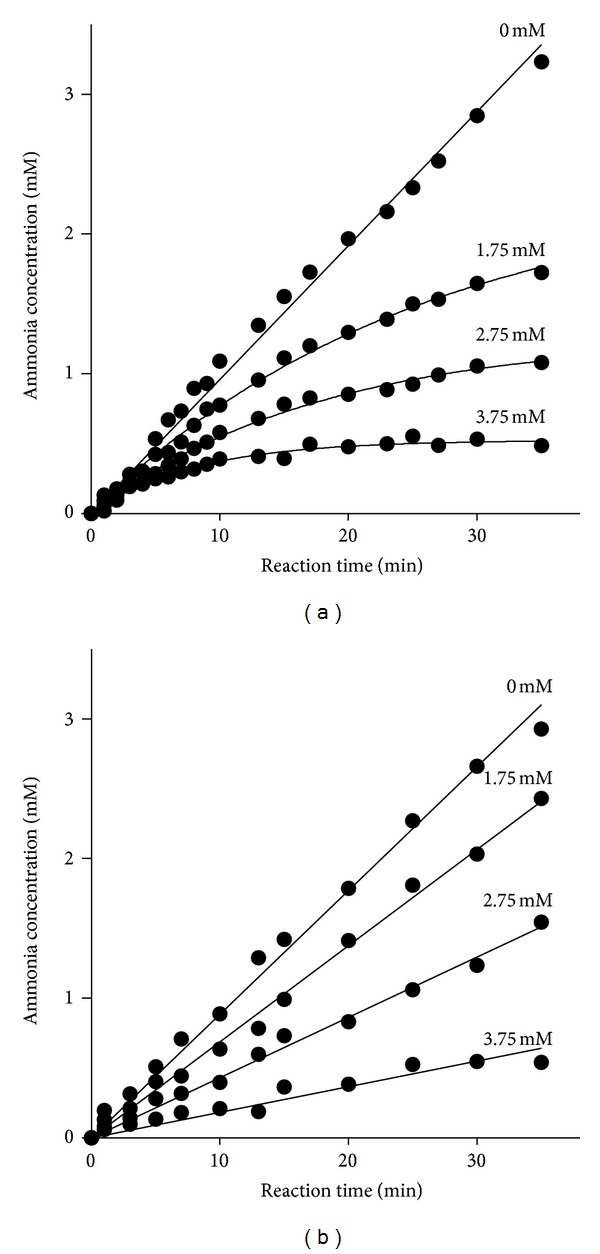
(a) Reaction progress curves of urease-catalyzed hydrolysis of urea in the presence of BA. (b) Steady-state analysis: concentration of ammonia versus time. BA concentration (mM) is numerically given.

The progress curves for urea hydrolysis under BA-inhibited urease catalyzation were shown in Figure 3. The reaction progress curves for the unpreincubated system were concave downward (Figure 4(a)), indicating that the velocity of urea hydrolysis decreased from an initial velocity (*v*_*o*_) to a much slower steady-state velocity (*v*_*s*_) according to the apparent first-order velocity constant *k*_app_. Such a behavior is characteristic of slow-binding inhibition elaborated by the theory of Morrison and Walsh [26]. The obtained results also showed that the initial velocity and the steady-state velocity were inhibitor-concentration-dependent. In terms of the preincubation system (steady-state analysis, Figure 4(b)), the linear curves proved that the reaction achieved the steady-state velocity (*v*_*s*_), being different from each studied inhibitor concentration.

The obtained relationship of the reaction velocities (*v*_*o*_; *v*_*s*_) *versus* the inhibitor concentration is characteristic of a two-step enzyme inhibitor interaction, mechanism *B* described as follows,

(2) $$\begin{array}{c} E+S\overset{k_{1}}{\underset{k_{2}}{⟷}}ES\overset{k_{7}}{\underset{}{\to }}E+P,\\ E+I\overset{k_{3}}{\underset{k_{4}}{⟷}}\underset{\text{slow}}{\underset{︸}{EI\overset{k_{5}}{\underset{k_{6}}{⟷}}EI^{*}}}, \end{array}$$

where *E* is enzyme, *S* is substrate, *P* is product, *I* is inhibitor, and *EI* and EI* are enzyme-inhibitor complexes, respectively. *k*_1_–*k*_7_ are velocity constants.

 Linear dependencies of 1/*v*_*o*_ and 1/*v*_*s*_ on the inhibitor concentration are used to evaluate the inhibition constants, *K*_*i*_ and *K*_i_*, as follows:

(3) $$\begin{array}{c} \frac{1}{v_{o}}=\frac{K_{M}}{v_{\max}S_{o}K_{i}}I+\frac{1}{v_{\max}}\left(1+\frac{K_{M}}{S_{o}}\right),\\ \frac{1}{v_{s}}=\frac{K_{M}}{v_{\max}S_{o}K_{i}^{*}}I+\frac{1}{v_{\max}}\left(1+\frac{K_{M}}{S_{o}}\right), \end{array}$$

where *K*_*M*_ is the Michaelis constant and *v*_max⁡_ is the maximum velocity given by the Michaelis-Menten equation for the uninhibited reaction; *S*_*o*_ denotes the initial concentration of urea; *K*_*i*_ and *K*_i_* are the inhibition constants defined as: *K*_*i*_ = [*E*][*I*]/[*EI*] and *K*_i_* = [*E*][*I*]/([*EI*] + [EI*]), respectively [26].

 By calculating from reciprocal dependence of *v*_*o*_ and *v*_*s*_ on the inhibitor concentration according to (3), it was found that the initial BA-urease complex formed rapidly with an inhibition constant of *K*_*i*_ = (3.89 ± 0.08) × 10^−3^ mM, followed by a slow isomerization into the final BA-urease complex with the overall inhibition constant of *K*_i_* = (1.47 ± 0.11) × 10^−4^ mM. The rate constant of the BA-urease isomerization indicated that forward process was rapid in contrast with slow reverse reactions. The overall inhibition constant obtained by the steady-state analysis was (1.32 ±0.16) × 10^−3^ mM. Furthermore, the shape of the curves in that case corresponded to the competitive slow-binding type of inhibition, as represented by (1). In details, the reaction was inhibited slightly in the initial period, characterized by high reaction rates *v*_*o*_. Then, in the later period, the inhibition became stronger, characterized by lower reaction rates *v*_*s*_. This indicated a competitive inhibition in both the initial and the steady-state stages of the inhibited reaction. 

 Taken together, the progress curves analysis and preincubation studies proved that the BA inhibition on urease was indeed in a slow-binding and competitive manner.

### 3.4. Urease Protection against BA Inactivation

By now, it has been found that there were two well-defined urease protectors, that is, the thiol-containing compounds (DTT, GSH, and L-cys) that interact with sulfhydryl groups of urease and the inorganic compounds (sodium fluoride and boric acid) reacting with active-site nickel ions [26, 27]. When equilibrated with the enzyme, the protectors by occupying the active site restrict the accessibility of inhibitions to the active-site functional groups [4]. Hence, both protectors were employed to investigate the inhibition target of BA.

**Figure 5:**
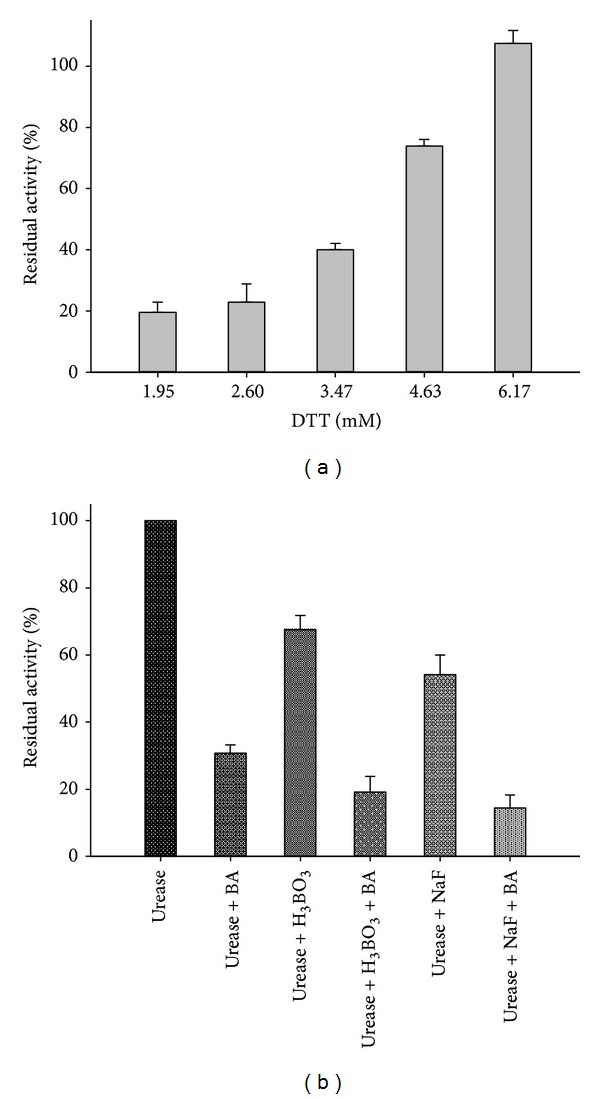
(a) DTT protection of urease against BA. Concentration of DTT (mM) is numerically given. (b) Protection of urease against BA inactivation by boric acid and fluoride. The results are expressed as means ± SD of the data from triplicate tests.

Figure 5(a) showed that the urease protection effect against inactivation by BA was enhanced as the concentration of thiol reagents increased. After the inactivation by 2.50 mM BA, DTT, a nucleophilic-reducing agent, could restore the urease activity in a concentration-dependent manner (6.17 mM or higher). This indicated that the thiol groups were exclusively involved in the inactivation of the enzyme and that there was a better affinity of BA towards DTT than the thiol group in urease. Likewise, in the protection experiments by GSH and L-cys, their protective potencies were found approximately three and two times, respectively, weaker than that of DTT (data not shown). By contrast, protections of sodium fluoride (a competitive slow-binding urease inhibitor) [27] and boric acid (a classical competitive urease inhibitor) [26–28] were insignificant.  Figure 5(b) demonstrated that, when urease was inactivated by BA in the presence of sodium fluoride and boric acid, the enzyme activity decreased to 15% and 20%, respectively, even lower than that in the presence of BA alone, suggesting a probable synergic relationship between BA and sodium fluoride or boric acid. 

 Taken together, better prevention by thiols than by inorganic compounds against BA inactivation indicated that the active-site sulfhydryl group is a residue responsible for urease inhibition.

### 3.5. BA-Thiol-Urease Interaction Test

**Figure 6:**
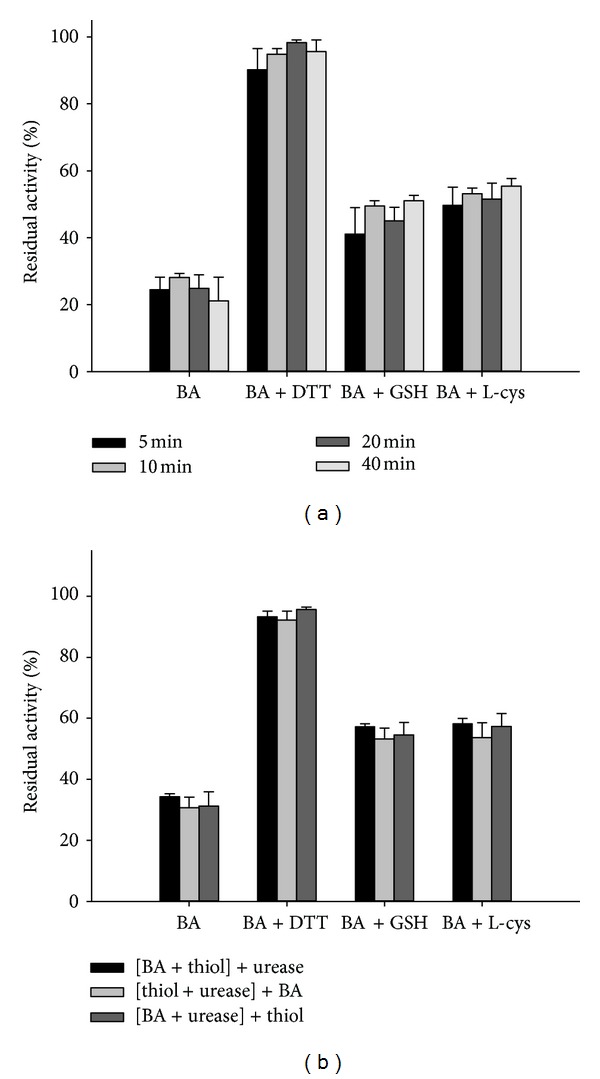
(a) Thiol influence on urease inactivation by BA relative to the control activity. The percent of the enzyme activity in the presence of BA without the thiol is given as comparison. Concentration of the thiol: L-cys, GSH, DTT, and BA were 3.75 mM. Enzyme activity was determined after 5, 10, 20, and 40 min of incubation. (b) Influence of thiol order of components preincubation on urease inactivation by BA. The initial 20 min preincubation mixture contained components given in brackets. The preincubation was continued the further 20 min after addition of the last component (component given outside for brackets). The final preincubation mixtures contained 0.25 mg/mL urease, 20 mM phosphate buffer, pH 7.0, 2 mM EDTA, 3.75 mM BA, and DTT, GSH, or L-cys. Enzyme activity was determined after 40 (20 + 20) min of preincubation time. The percent of the enzyme activity in the presence of BA without the thiol is given for comparison. The results are expressed as means ± SD of the data from triplicate tests.

The role of thiols in BA inactivation was studied by comparing urease activities in thiol-free system at four time points of incubation. It was found that monothiol (L-cys or GSH) and dithiol (DTT) could alleviate the inactivation by BA, and urease remained highly active in spite of BA presenting in the incubation mixture; when the thiol-containing compounds provided thiol groups, concentration was equal or higher than that of BA (Figures 6(a) and 6(b)). However, incubation time had no significant effect on the BA-thiol-urease interaction. And the protection potency did not matter with the addition order of urease, inhibitors, and protectors. 

The presence of the thiol-protector in the incubation system allowed BA to react with thiols from the urease and those in the “free” thiol-protector. The thiols presenting in the protein were much less reactive than those presenting externally in the form of L-cys, GSH, or DTT. The decreases of urease activity in the thiol-free system and system with the thiols were compared, suggesting that the general losses of urease activity in both systems remained, but it was slowed down in the presence of thiols, especially in the presence of DTT. These data suggested that BA-thiol interaction was strategic for the inactivation rate decrease. 

### 3.6. Reactivation of BA-Inactivated Urease

**Figure 7:**
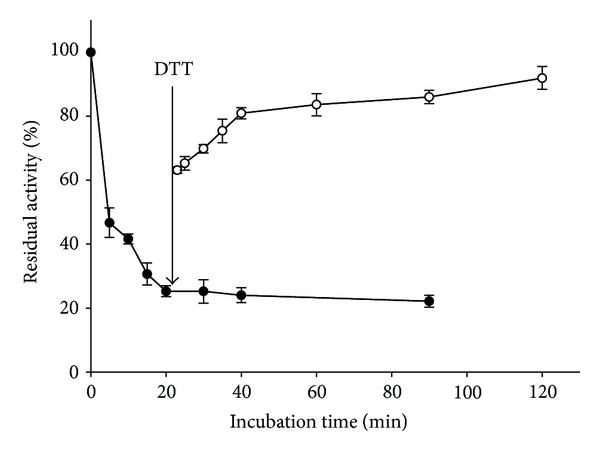
Reactivation of BA-inactivated urease by DTT. Activity of urease inactivated by BA (*⚫*) and after adding DTT (○). Urease was inactivated by 3.75 mM BA, and 3.75 mM DTT was added into the reaction system 20 min later (as indicated by the vertical arrow). The results are expressed as means ± SD of the data from triplicate tests.

To investigate whether the inactivation of urease by BA is reversible, the reactivation of BA-inactivated urease was carried out in two ways. In the first way, by addition of DTT after the 20 min incubation of urease with BA, urease activity recovered in a time-dependent manner: after 1.5 h, the enzyme had restored ca. 90% of its initial activity (Figure 7). After reactivated by DDT, retreatment of BA could not inhibit the urease activity again. This evidence indicated that the urease-BA complex was less resistant for chemical approach.

 By contrast, in the second way, by multidilution, it was shown that urease remained in constant activity as the concentration of ammonia increased, which indicated that an insignificant amount of the active enzyme separated from urease-BA complex after dilution because of no further active urease releasing. Taken together, there would be a supposed reversibility between urease and BA, in which the chemical approach but not multidilution could recover the enzyme activity that had been inactivated by BA.

### 3.7. Molecular Docking

**Figure 8:**
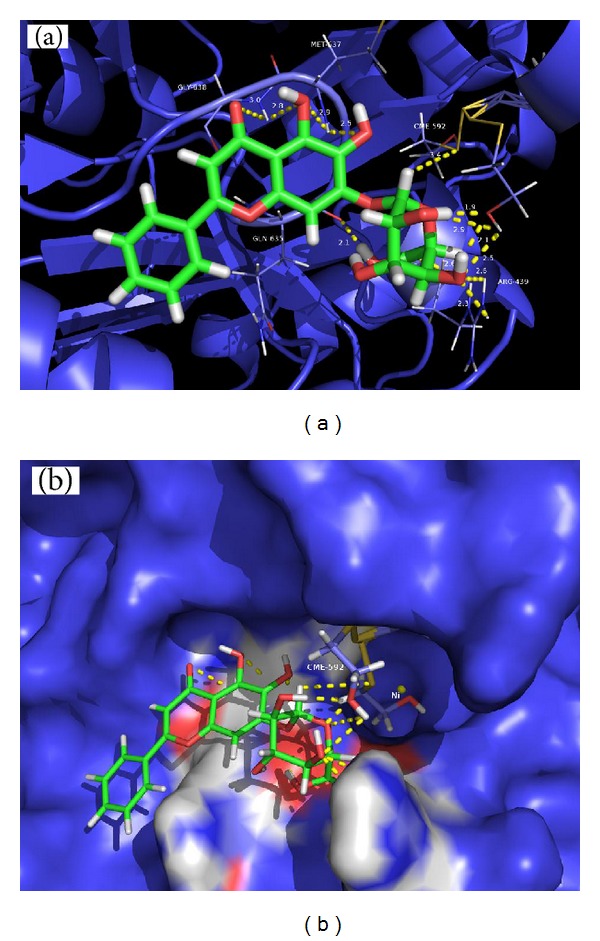
(a) Molecular docking simulations obtained at the lowest energy conformation, highlighting potential hydrogen contacts of BA (colored by atom: carbon is green; nitrogen is blue; oxygen is red; hydrogen is white; sulfur is yellow). For clarity, only interacting residues are labeled. Hydrogen bonding interactions are shown by dashes. These figures were created using PyMol. (b) Surface representation of the active-site flap of the jack-bean urease with BA shown at the entrance of the binding pocket.

In an effort to elucidate the inhibition mechanism revealed by the kinetics study, molecular docking of BA into the crystal structure of jack-bean urease (3LA4 in the Protein Data Bank) was performed by the AutoDock program, and the best possible binding modes were shown in Figure 8. In the best possible binding mode, BA tightly anchored the helix-turn-helix motif over the active-site cavity through O−H•••S, N−H•••O, and O−H•••O hydrogen bonding interactions. This mode made BA engage a cleft beside the active-site cavity, using 13 typical hydrogen bonds to anchor the flap tightly with the backbone of the enzyme, thereby preventing the flap from backing to the close position. 1′′-OH in the saccharide group of BA as the hydrogen bond donor was found between the OH and the backbone S atom of CME 592 (H•••S distance = 3.4 Å), which was located on the mobile flap closing the active site of the enzyme. 4′′-OH of the saccharide group in BA formed a strong O−H•••O hydrogen bond (H•••O distance = 2.1 Å) and a strong hydrogen bond (H•••O distance = 1.9 Å) to the backbone oxygen and hydrogen atom of Cys-592 (marked as CME 592), respectively. And 6′′-OH of BA was involved in the interactions considered as hydrogen bonds between the H atom and the backbone CO group of GLN635 with an O•••H bond length of 2.1 Å. In addition, the 4′′-OH of BA was bound via two hydrogen bonds to NH_2_ of ARG439 with O•••H distance of 2.3 Å and 2.6 Å, respectively. 6-OH and 6′′-O in saccharide group of BA as acceptor accepted one hydrogen bond from NH group of MET637 with O•••H distance of 2.5 Å and from NH group of ARG439 with an O•••H distance of 2.6 Å, respectively. 

The Cys-592 (marked as CME 592) is a key residue located at the mobile flap covering the active site, one per each of the six sites in the hexameric molecule [29]. Besides being directly involved in the architecture of the active site, the residue has a vital role in positioning other key residues in the active site appropriately for the catalysis [30, 31]. The flexible flap goes through an open-closed-open procedure, effectively activating the inert urea leading to activated enzyme during the normal urea catalyzed by urease [32]. And modification of Cys-592 resulted in restriction of the mobility of the flap, subsequently perturbed reaction, and reduced enzyme activity. Moreover, other residues of the flap at the entrance of the binding pocket, such as GLY638, MET637, GLN635, and ARG439, participate in the substrate binding, stabilize the catalytic transition state, and accelerate the reaction mainly through hydrogen bonding. It was reported that some urease inhibitors depressed jack-bean urease activities by interacting with the sulfhydryl group of residues, especially the Cysteine-592 [4].

As the results depicted, BA possibly made hydrogen bonding interactions with the side chains of the abovementioned residues, especially the active-site flap Cys-592, hence preserving the flap in an open conformation and resulting in an inactivation. The observations were soundly supportive of the earlier conclusion drawn from the urease protection experiments performed with the active-site binding inhibitors, which substantiated the fact that inhibition by BA was by the way of destroying the participation of sulfhydryl group of the active-site cysteine. Taking into account the peculiarities of the active-site flap cysteine in the urease catalysis and sulfhydryl group in urease activity, it can be inferred that BA made contacts with the side chains of cysteine residues, especially sulfhydryl group, which was reflected in their enhanced affinity to the Cys-592 residues. As a result, the mobility of flap was restricted, and, finally, the enzymatic activity was significantly declined.

## 4. Conclusion

According to the systematic investigation on the kinetics and mechanism of the urease inhibition by BA in the present study, it could be deduced that BA was a competitive inhibitor targeting thiol groups in the active site of urease in a slow-binding manner, both reversibly and concentration dependently, serving as a promising urease inhibitor for treatments of the urease-related diseases.
